# Preferences for and drivers of adult vaccination clinic site selection: A cross-sectional study in 30 provinces in China

**DOI:** 10.1080/21645515.2024.2442104

**Published:** 2025-01-10

**Authors:** Yuxi Liu, Yanlin Cao, Yugang Li, Siyuan Liu, Yunshao Xu, Weizhong Yang, Luzhao Feng

**Affiliations:** a School of Population Medicine and Public Health, Chinese Academy of Medical Sciences and Peking Union Medical College, Beijing, China; b State Key Laboratory of Respiratory Health and Multimorbidity, Key Laboratory of Pathogen Infection Prevention and Control (Peking Union Medical College), Ministry of Education, Beijing, China

**Keywords:** Vaccine, non-NIP vaccine, vaccination site, vaccination preferences, vaccine hesitancy

## Abstract

Focusing on vaccines available to adults and not in the immunization schedule, this study investigates the preferences and factors influencing adults in selecting vaccination clinic locations. It aims to provide strategic insights for boosting vaccination rates by analyzing adults’ decision-making factors. This contributes to developing more efficient, patient-focused vaccination strategies that tackle vaccine hesitancy and improve access to vaccination sites. We conducted a cross-sectional study through the “YueMiao” platform from November 1 to December 10, 2023, using convenience and purposive sampling to engage 2014 participants. We collected data via online surveys that included questions about sociodemographic characteristics, sources of vaccination clinic information, clinic satisfaction, and the impact of site selection on vaccination decisions. Our findings reveal that adults’ site preferences for vaccination are influenced by gender, age, income, and vaccination history. Participants showed a strong preference for locations that offer convenience, efficiency, transparent pricing, and a comfortable environment. Analysis of service satisfaction at these clinics indicates that vaccinated individuals report higher satisfaction with appointment systems, wait times, and service hours than those unvaccinated. Furthermore, the preference for vaccination sites consistently aligns with the vaccine type, with a majority opting for community health service centers. Our results suggest that public health strategies should concentrate on enhancing site convenience, service quality, and information transparency to elevate adult vaccination rates. Future initiatives should aim to increase public trust in vaccines, improve the selection and quality of vaccination sites, and effectively utilize digital technology for spreading vaccination information.

## Introduction

Globally, pathogens such as the influenza virus, streptococcus pneumoniae, respiratory syncytial virus (RSV), and varicella-zoster virus pose a serious challenge to public health and life safety. Such pathogens not only threaten individual lives, but also seriously affect people’s mental health and quality of life. In China, a model study based on national influenza surveillance and cause of death surveillance data estimated that from 2010–2011 to 2014–2015, there was an average of 88,000 (95% CI: 84000–92,000) excess deaths from influenza-related respiratory diseases each year.^1^ The severity of the disease burden is not only reflected in excess deaths, but also includes long-term disability caused by the disease, economic losses to families and society, and high demands on medical resources. According to data from the CDC, hepatitis B, as a serious blood-borne disease, can cause major liver disease, including fatal cancers.^2^ Further, a study showed that the economic burden of vaccine-preventable diseases among adults approximates $27 billion per year.^3^ In addition, the average age of incidence of cervical cancer is gradually decreasing. The average age of patients diagnosed with cervical cancer between 2000 and 2014 decreased from 53.48 years to 50.32 years, and the proportion of people aged 30–64 years increased from 67.21% to 84.48%.^4^

The WHO’s ‘Immunization Agenda 2030’ emphasizes the ambitious goal of achieving global health coverage through vaccines.^5^ Vaccination significantly reduces infectious disease burden,^6–8^ and adult vaccines for influenza,^9,10^ herpes zoster,^11^ and pneumococcal diseases^12,13^ have been recognized for their global effectiveness and safety.^14,15^ Despite strong evidence supporting vaccination, adult coverage remains below public health targets worldwide,^16,17^ exposing the challenges public health systems encounter in bolstering adult vaccination. The decision-making process for adult vaccination is multifaceted^18^ and is influenced by personal beliefs, socioeconomic factors, historical vaccination experiences,^19^ and healthcare provider recommendations.^20^ Selecting accessible and convenient vaccination sites, such as local pharmacies and community centers, is crucial for improving vaccination rates^21^ by enhancing trust, especially among those hesitant or mistrustful of the healthcare system.^22^

The ‘YueMiao’ platform is an adult disease prevention service platform offering vaccine appointment services, disease prevention information, as well as cervical and breast cancer screening services. As of 2024, ‘YueMiao’ has expanded to cover 30 provincial-level regions in China, 245 prefecture-level cities, and 1,193 counties, connecting over 6,000 vaccination service institutions and serving more than 42 million users. The platform simplifies the vaccination appointment process and enhances disease prevention management through big data analysis. This study uses data collected from YueMiao users to identify barriers to vaccination decisions, with particular focus on the selection of adult vaccination clinic sites. The findings aim to provide data-driven insights to inform the development of policies and strategies that better meet the needs of diverse populations, particularly for vaccines outside of national immunization programs.

## Methods

### Study participants and survey design

The questionnaire was distributed through the YueMiao platform. Participants accessed the YueMiao app, where they were presented with a pop-up containing a QR code for the survey. Users voluntarily scanned the QR code, which redirected them to the Wenjuanxing platform (Changsha Haoxing Information Technology Co., Ltd., Changsha, China) to complete the questionnaire. All data collection occurred on the Wenjuanxing platform, ensuring a streamlined and secure survey process. The individuals who refused to complete the questionnaire exited the survey site automatically. The sample size was estimated by the formula:

$$N=\frac{Z_{a}^{2}\times p\times \left(1-p\right)}{\delta^{2}}$$

based on 5% type one error (a = 0.05), with the assumed proportion of adults willing to take the adult vaccines (p) set at 30%, and the maximum permissible error (δ) defined as 0.1 ×p. Based on these parameters, the required sample size was calculated to be 1,067 participants. The survey was conducted from November 1 to December 10, 2023. This period was chosen because it corresponds to a period of high activity on the YueMiao platform, which helped ensure the representativeness of the sample and increased participation rates.

### Survey content

A cross-sectional survey design was used to collect data on 1) sociodemographic characteristics, 2) channels for obtaining vaccination clinic information and clinic satisfaction, 3) willingness to choose vaccination sites and awareness of vaccination clinics, and 4) the impact of vaccination site choice on vaccination decision-making.

### Quality control

All data provided to the investigators were de-identified, and the participants were anonymous. Quality control was conducted through the survey platform, wherein the respondents were invited to complete the questionnaire voluntarily. Each respondent, identified by the same device, username, and IP address, could fill in the questionnaire only once. After the responses were submitted, each respondent was screened by the investigators to retain the valid questionnaires. Those that failed the attention check, provided the same answer for all questions or provided cyclical responses, who finished the questionnaire within 60 s, and those <18-years-old were removed from the dataset. Additionally, consistency between the IP address and the selected region was verified to filter out random answers. After eliminating invalid responses, 2,014 valid questionnaires remained for analysis.

### Statistical analysis

The data were imported into Microsoft Excel and auto-populated using the Questionnaire Star Online Survey Management System. Statistical analyses were performed using R version 4.3.1 and STATA version 14.2 (Stata Corp., College Station, Texas, USA). Categorical data are presented as frequencies and percentages (%). The associations between sociodemographic factors and choice of vaccination site were analyzed using univariate logistic regression. The chi-square ($\chi^{2}$) test was applied to test for differences in the proportion of satisfaction with vaccination clinics between individuals vaccinated against influenza and those not vaccinated during the 2023 influenza season. Factors influencing beliefs about influenza vaccination were identified using univariate and multivariate logistic regression analyses. The results were expressed as odds ratios (ORs) with 95% confidence intervals (CIs) and adjusted odds ratios with 95% CIs. A two-tailed test approach was employed, with the significance level (α) set at 0.05.

## Results

### General information

The characteristics of the study participants are summarized in Table 1. Of the 2,014 participants, most were women, with a concentration of individuals below the age of 44 years. The distribution of per capita disposable income among participants was relatively equitable, and a significant proportion of participants had attained an educational level of a bachelor’s degree or higher. There was a marked preference for vaccinations at community health service centers over secondary and tertiary medical facilities. However, vaccination rates for influenza, herpes zoster, and pneumococcal vaccines were low during the specified intervals.

**Table 1.** Participants’ characteristics and vaccine site preference (*n* = 2,014).

Characteristics	N	%
Per capita disposable income^a^		
Low	210	10.4
Moderate	898	44.6
High	906	45.0
Gender		
Male	153	7.6
Female	1861	92.4
Age (years)		
18–24	559	27.8
25–34	739	36.7
35–44	611	30.3
≥45	105	5.2
Educational background		
Junior high school and below	281	14.0
High school or equal	428	21.3
Bachelor or equal	1168	58.0
Master and above	137	6.8
Average monthly household income (CNY)		
<3000	369	18.3
3000–5000	652	32.4
5001–8000	445	22.1
8001–10000	207	10.3
>10000	341	16.9
Suffering from chronic disease		
Yes	139	6.9
No	1875	93.1
Preferred vaccination site		
Community health service centers and other sites	1454	72.2
Secondary and tertiary hospital	560	27.8
Vaccinated with adult vaccines		
Never vaccinated	60	3.0
Only vaccinated with the COVID-19 vaccine	714	35.5
Vaccinated with other vaccines^b^	1240	61.6
Influenza vaccinated between August and December 2023		
Yes	240	11.9
No	1774	88.1
Herpes zoster vaccinated		
Yes	132	6.6
No	1882	93.4
Pneumococcal vaccinated between 2019 and 2023		
Yes	177	8.8
No	1837	91.2

^a^In terms of per capita disposable income, the provinces were divided into three levels: low, moderate, and high. Low for Jilin, Shanxi, Heilongjiang, Henan, Guangxi, Xinjiang, Qinghai, Guizhou, Tibet, Yunnan, and Gansu; moderate for Inner Mongolia, Chongqing, Hunan, Anhui, Hubei, Jiangxi, Shaanxi, Hainan, Hebei, Sichuan, and Ningxia; high for Beijing, Shanghai, Tianjin, Jiangsu, Zhejiang, Fujian, Guangdong, Shandong, Liaoning, Hong Kong, and Taiwan.

^b^Other vaccines include HPV vaccine, influenza vaccine, hepatitis B vaccine, herpes zoster vaccine, pneumococcal vaccine, hepatitis A vaccine, measles-mumps-rubella vaccine, and hepatitis E vaccine.

**Figure 1.**
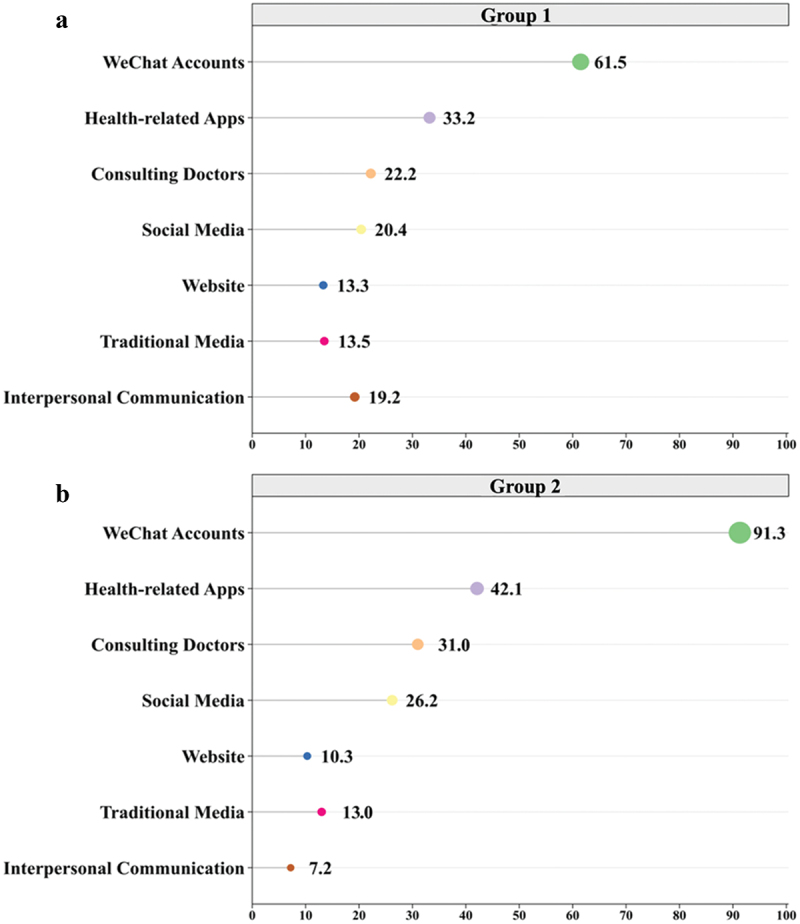
Proportions of different information channels for vaccination clinics: (a) known channels of Acquisition; (b) preferred channels of acquisition.

Figure 1 shows a comparison of the current and expected sources of vaccination clinic information. WeChat was the top source, followed by health apps and doctor consultations. Social media, websites, traditional media, and interpersonal communications were used less frequently. Future expectations showed a significant rise in WeChat use (91.3%), an increased preference for health apps (42.1%), and a desire for more direct information from doctors (31.8%). Social media, websites, traditional media, and interpersonal communication were less frequently utilized, denoting them as ancillary sources of information.

### Primary factors influencing the selection of preferred vaccination locations

Regarding demographic characteristics, sex significantly influenced the choice of vaccination site in univariate analysis (Table 2). Female participants were more likely than their male counterparts to opt for vaccination in secondary and tertiary hospitals. Age played a consistently significant role in the selection process for vaccination sites, with the age group of 35–44 years demonstrating a pronounced preference for community health service centers. Furthermore, families with a monthly income exceeding 10,000 yuan showed a greater preference for community health service centers than those in the lower-income bracket, suggesting income as a potential determinant in vaccination site selection. A history of chronic disease did not significantly impact site preference. Vaccination history emerged as a crucial determinant; individuals with a history of receiving vaccines other than the COVID-19 vaccine were more inclined to receive vaccinations at community health service centers.

**Table 2.** Logistic regression analysis of the determinants of anticipated vaccination locations.

Characteristics	Community health service centres/other vaccination sites	Secondary/tertiary hospitals		
	*n* = 1454	*n* = 560		
	N (%)	N (%)	*p*	OR 95% CI)
Per capita disposable income				
Low	146 (10.0)	64 (11.4)		ref
Moderate	631 (43.4)	267 (47.7)	.83	0.97 (0.70, 1.34)
High	677 (46.6)	229 (40.9)	.12	0.77 (0.55, 1.07)
Gender				
Male	122 (8.4)	31 (5.5)		ref
Female	1332 (91.6)	529 (94.5)	.03	1.56 (1.04, 2.35)
Age				
18–24	352 (24.2)	207 (36.9)		ref
25–34	537 (36.9)	202 (36.1)	<.01	0.64 (0.51, 0.71)
35–44	478 (32.9)	133 (23.8)	<.01	0.47 0.37, 0.61)
≥45	87 (6.0)	18 (3.2)	<.01	0.35 (0.21, 0.60)
Educational background				
Junior high school and below	197 (13.6)	84 (15.0)		ref
High school or equal	321 (22.1)	107 (19.1)	.15	0.78 (0.56, 1.09)
Bachelor or equal	825 (56.7)	343 (61.3)	.86	0.98 (0.73, 1.30)
Master and above	111 (7.6)	26 (4.6)	.02	0.55 (0.33, 0.90)
Average monthly household income (CNY)				
<5000	704 (48.4)	317 (56.6)		ref
5000–10000	479 (32.9)	173 (30.9)	.05	0.80 (0.64, 1.00)
>10000	271 (18.6)	70 (12.5)	<0.01	0.57 (0.43, 0.77)
History of chronic disease				
Yes	100 (6.9)	39 (7.0)		ref
No	1354 (93.1)	521 (93.0)	.95	0.99 (0.67, 1.45)
Vaccinated with adult vaccines				
Only vaccinated with the COVID-19 vaccine/never vaccinated	538 (37.0)	236 (42.1)		ref
Vaccinated with other vaccines	916 (63.0)	324 (57.9)	.03	0.81 (0.66, 0.98)

**Figure 2.**
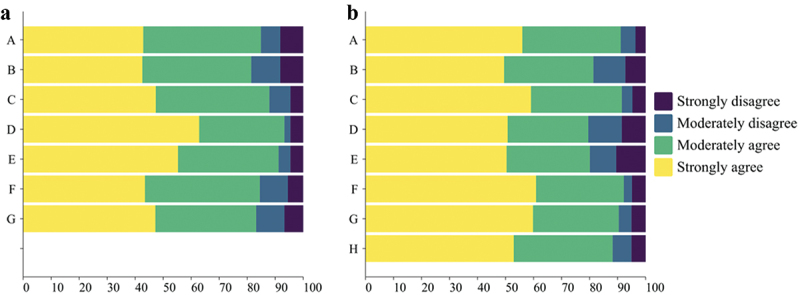
Factors influencing the selection of different vaccination sites among study participants. (a) Participants selecting community health service centres (*n* = 1,454); (b) participants selecting vaccination clinics within secondary and tertiary hospitals (*n* = 560).Annotations: A. Vaccination recommended by a physician during consultation; B. Transparent and comparatively lower vaccine prices than at other sites; C. Spacious and well-maintained vaccination environment; D. Proximity of vaccination site and reduced transportation expenses; E. Efficient vaccination process with minimal waiting periods; F. Enhanced management of adverse reactions; G. Greater vaccine availability than at alternative sites; H. Convenience of receiving vaccination post-consultation.

Participants indicated their reasons for choosing the vaccination sites (Figure 2). Those favoring secondary and tertiary hospitals valued spacious environments, superior adverse reaction management, and larger vaccine stocks. Those who preferred community health center prioritized proximity, lower transport costs, efficient processing, and shorter wait times. Perceptions of transparent and affordable vaccine pricing were present in both groups, with marginally higher percentages in the secondary and tertiary hospital groups. The convenience of receiving a vaccine following medical consultation in relation to the clinical functions of the hospital was not identified as a major factor in participants’ choice of vaccination location.

### Satisfaction analysis of vaccination clinic services

The survey targeted 240 participants who had been vaccinated against influenza from August 2023 to the time of questionnaire submission, alongside 1,774 individuals who had not been vaccinated (Table 3). All participants, regardless of their vaccination status, were recruited through the YueMiao platform. In the comparison of clinic service satisfaction between vaccinated and unvaccinated participants, the vaccinated group exhibited significantly lower proportions of dissatisfaction in the areas of appointment scheduling, duration of queuing or waiting, awareness of service operational hours, and knowledge of channels for obtaining vaccination information. Conversely, no significant differences were observed between the vaccinated and unvaccinated groups in dissatisfaction levels regarding vaccination procedures and reminders for subsequent doses as well as the cleanliness of the vaccination environment.

**Table 3.** Satisfaction with service at vaccination clinics: comparison of individuals vaccinated and not vaccinated for Influenza^a^.

Category	Vaccinated	Unvaccinated	χ^2^	*p*
	*n* = 240	*n* = 1774		
	N (%)	N (%)		
Satisfaction with vaccine clinic location	209 (87.1)	1498 (84.4)	1.14	.29
Satisfaction with appointment scheduling	211 (87.9)	1458 (82.2)	4.89	.**03**
Satisfaction with waiting time at vaccine clinic	198 (82.5)	1220 (68.8)	19.12	**<.01**
Satisfaction with vaccination procedure and reminder information	199 (82.9)	1419 (80.0)	1.15	.28
Satisfaction with vaccination environment	217 (90.4)	1529 (86.2)	3.27	.07
Awareness of service hours	179 (74.6)	1123 (63.3)	11.77	**<.01**
Knowledge of vaccination information channels	189 (78.8)	1157 (65.8)	17.46	**<.01**

^a^Status of influenza vaccination from August to December 2023.

### Impact of vaccination site selection on vaccine uptake decisions

**Figure 3.**
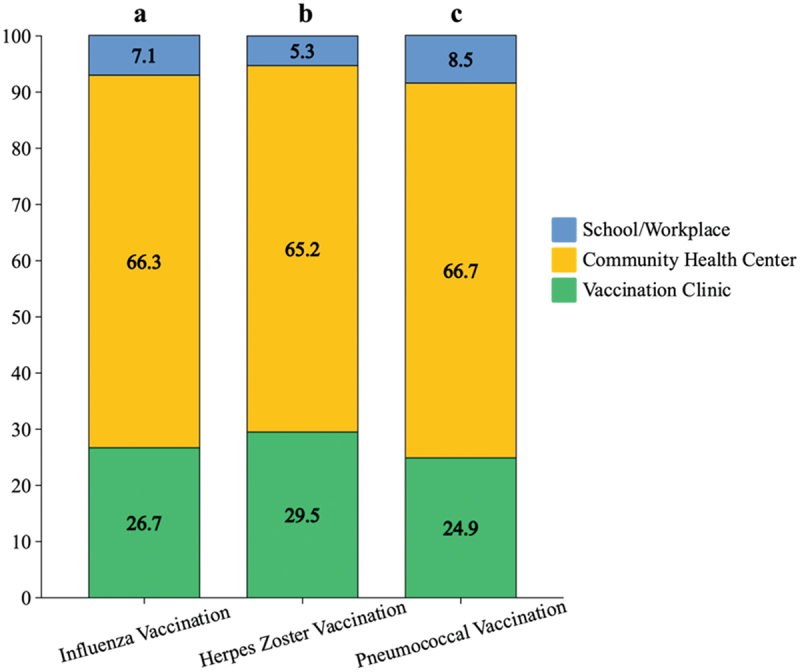
Percentage of site preference for different vaccinations. a. Vaccinated with the influenza vaccine from August to December 2023 (*n* = 240); b. Previously vaccinated with the herpes zoster vaccine (*n* = 132); c. Received the pneumococcal vaccine between 2019 and 2023 (*n* = 177).

The choice of the vaccination location for influenza, herpes zoster, and pneumococcal vaccines showed a consistent pattern (Figure 3). From August to December 2023, 240 individuals (11.9%) were vaccinated against influenza, 132 (6.6%) had previously received the herpes zoster vaccine, and 177 (8.8%) had been vaccinated against pneumococcal pneumonia within the past five years. The vaccination sites included clinics at secondary and tertiary hospitals, community health service centers, and educational or workplace settings. Community health service centers were preferred for vaccination services by most participants, although a portion of the population also selected clinics at secondary and tertiary hospitals.

Focusing on the influenza vaccine administration from August to December 2023, this study explored how factors such as the perceived importance of the influenza vaccine, cost considerations, convenience, physician advice, knowledge of vaccination sites, and information acquisition channels affect influenza vaccination decisions (Table 4). Multivariate analysis revealed a higher likelihood of vaccination among those who valued the vaccine’s importance, were unconcerned about service fees, were influenced by convenience, and received physician recommendations. Awareness of community health centers over hospital clinics and a preference for secondary and tertiary hospitals were also correlated with increased vaccination willingness. Knowledge of vaccination clinic information channels positively affected vaccination rates, whereas familiarity with service hours showed a positive but not significant trend toward vaccination intent.

**Table 4.** Logistic regression analysis of factors influencing decision making for influenza vaccination.

	Vaccinated	Unvaccinated	Univariate Logistic Regression		Multivariate Logistic Regression	
	*n* = 240	*n* = 1774				
Factor	N (%)	N (%)	*p*	OR (95% CI)	*p*	OR (95% CI)
Importance of adult influenza vaccination						
Not important	12 (5.0)	157 (8.9)		ref		ref
Moderately important	13 (5.4)	122 (6.9)	.43	r0.72 (0.32, 1.63)	.31	0.65 (0.28, 1.50)
Very important	215 (89.6)	1495 (84.2)	.**04**	0.53 (0.29, 0.97)	.**05**	0.53 (0.29, 0.99)
Does the vaccine service fee influence your selection?						
Yes	85 (35.4)	765 (43.1)		ref		ref
No	155 (64.6)	1009 (56.9)	.**02**	0.72 (0.55, 0.96)	**<.01**	0.63 (0.46, 0.86)
Does vaccination accessibility influence your choice?						
Yes	106 (44.2)	621 (35.0)		ref		ref
No	134 (55.8)	1153 (65.0)	**<.01**	1.47 (1.12, 1.93)	**<.01**	1.58 (1.16, 2.16)
Does medical recommendation impact your vaccination decision?						
Yes	89 (37.1)	504 (28.4)		ref		ref
No	151 (62.9)	1270 (71.6)	**<.01**	1.49 (1.12, 1.97)	.**03**	1.42 (1.04, 1.93)
Known vaccination locations						
Community health service center	165 (68.8)	1098 (61.9)		ref		ref
Hospital clinic	54 (22.5)	557 (31.4)	**<.01**	1.55 (1.12, 2.14)	**<.01**	1.93 (1.35, 2.75)
Organised by workplace/school	18 (7.5)	77 (4.3)	.11	0.64 (0.37, 1.10)	.25	0.71 (0.40, 1.28)
Unclear	3 (1.2)	42 (2.4)	.22	2.10 (0.64, 6.87)	.30	1.90 (0.57, 6.37)
Preferred vaccination locations						
Secondary/tertiary hospital	80 (33.3)	466 (26.2)		ref		ref
Community health service center	136 (56.7)	1170 (66.0)	.**01**	1.48 (1.10, 1.99)	**<.01**	1.88 (1.36, 2.60)
Workplace/school	24 (10.0)	138 (7.8)	.96	0.99 (0.60, 1.62)	.46	1.23 (0.71, 2.11)
Awareness of vaccination clinic operational hours						
Yes	179 (74.6)	1123 (63.3)		ref		ref
No	61 (25.4)	651 (36.7)	**<.01**	1.70 (1.25, 2.31)	.19	1.29 (0.88, 1.89)
Knowledge of vaccination information sources						
Yes	189 (78.7)	1157 (65.2)		ref		ref
No	51 (21.3)	617 (34.8)	**<.01**	1.98 (1.43, 2.73)	.**01**	1.65 (1.11, 2.47)

## Discussion

Our findings highlighted that adults prefer community- and hospital-based clinics for vaccination, underscoring their trust in the healthcare system. These findings confirm the established claims that sociodemographic variables, such as age,^23^ education, and income,^24^ significantly influence health behaviors, including vaccination. This consistency with previous research highlights the need for customized public health strategies that consider the diverse backgrounds and needs of adults. Simultaneously, the current study highlighted the profound influence that healthcare providers have on guiding vaccination decisions. Viewing trust in healthcare professionals as a cornerstone of vaccine acceptance illuminates the critical need for clear and effective communication between healthcare providers and patients regarding vaccination. Visual presentation and social media platforms, such as Twitter, have come to play a vital role in widely disseminating prevention messages.^25^ The critical role of digital and traditional information channels in shaping vaccination behavior was evident in our analysis. The shift to digital media as the primary source of health information demonstrates that public health campaigns must adapt to these changing dynamics to improve vaccine knowledge and counter misinformation.^26^ The decision-making process regarding vaccination site selection is influenced by multiple factors,^27^ including logistical considerations such as proximity to home or work, appointment availability, and wait times. These factors, combined with personal health beliefs and prior experience of healthcare providers, highlight the complexity of choosing vaccination sites. Our findings suggest that public health strategies should be adaptable, accessible, and responsive to adults’ diverse needs. Additionally, improving the accessibility and convenience of vaccination sites, such as community health centers and medical facilities, and enhancing the safety and credibility of vaccination in non-traditional settings, such as pharmacies^28,29^ or workplace clinics, can address logistical barriers and expand vaccine access.

The practical implications of this study for public health strategies are diverse. By understanding the preferences and needs of the adult population, policymakers and health practitioners can design vaccination programs that are not only effective but also meet the expectations of various groups. Improving access to vaccination sites is a key strategy. Internationally, an array of strategies have been implemented to augment adult vaccination rates. In the United States, adults are afforded a multitude of vaccination venues, ranging from medical facilities to pharmacies and community vaccination centers, all designed to cater to the varied needs of the public with convenient solutions.^30^ The United Kingdom capitalizes on its National Health Service to administer vaccinations across a spectrum of medical settings – hospitals, general practitioner clinics, and community health centers – to ensure the ubiquity and accessibility of vaccination services.^31^ European nations have uniformly enacted definitive policies and guidelines advocating adult vaccination and have made us of a wide selection of medical venues to facilitate vaccination, thus fostering increased coverage.^32^ Japan has emphasized the provision of accessible vaccination services nationwide to ensure that adults can readily acquire the vaccines necessary to safeguard public health and mitigate disease transmission.^33^ China has improved access to and accessibility of vaccination services by incentivizing the establishment of vaccination clinics in secondary and tertiary hospitals.^34^ Moreover, by enhancing public awareness through health education campaigns^35^ and tracking vaccination status through electronic health records,^36^ China has markedly improved the efficacy and efficiency of its vaccination initiatives. Effective public health interventions should aim to close the gap between vaccine supply and vaccination by adopting approaches that meet the specific preferences and needs identified in this study. This includes leveraging trust in healthcare providers to combat vaccine hesitancy, optimizing the use of digital and traditional information channels to reach a wider audience, and ensuring that vaccination sites are convenient, popular, and efficient.

While the YueMiao platform provides a convenient channel for collecting data on adult vaccination behaviors, the generalizability of the findings is limited by the platform’s user demographics. The platform’s users are primarily urban, digitally literate, and younger, which may not fully represent the broader adult population in China. Our sample consisted of a higher proportion of women, younger individuals, and those with higher income and education levels. According to the 2023 National Report on the Development of Aging Affairs,^37^ approximately 21.1% of the population is aged 60 or older, yet this group was underrepresented in our sample, potentially introducing bias in understanding vaccination behaviors among older adults, a key target group for many vaccines like influenza and pneumococcal vaccines. However, focusing on this specific group also has distinct advantages. The higher digital engagement and urban background of the platform’s users provide valuable insights into vaccination preferences and behaviors, particularly in urban areas where digital health platforms facilitate more convenient appointment scheduling and access to vaccination information. The behavior of this group reflects the practical impact of digital health strategies and public health interventions, which is critical for the promotion of digital health platforms in urban regions. Future studies should aim to include a broader sample that encompasses rural and lower-income populations to gain a more comprehensive understanding of vaccination behaviors across diverse groups. Additionally, self-reported data may introduce response bias, and future research could benefit from incorporating objective data sources, such as medical records, to minimize such bias.

Drawing on the comprehensive findings of our study, we propose a set of policy recommendations to optimize immunization services and enhance vaccine uptake among adults. Central to these recommendations is the training of healthcare providers in effective communication strategies that not only convey factual information about the benefits and safety of vaccines but also address common concerns and misinformation in a respectful and empathetic manner. This communication effort should be supported by improving both the physical and digital accessibility of vaccination services. For example, leveraging mobile health applications (mHealth), such as the YueMiao platform, to provide personalized reminders and facilitate vaccine appointments has proven effective in increasing vaccination rates, especially by reducing missed appointments.^38^ Digital platforms like WeChat have also shown great potential in disseminating accurate vaccination information and appointment booking services, leading to greater public engagement and vaccination uptake.^39^ Additionally, our findings highlight the need for tailored public health messaging that addresses the specific cultural, socioeconomic, and psychological factors that influence vaccine decision-making within diverse demographic groups. This could include segmenting target audiences and developing customized communication that resonates with their unique concerns, supported by digital tools that ensure convenience and transparency in accessing vaccination services.^40^ By combining these targeted communication strategies with enhanced access through digital and physical infrastructure, we can significantly reduce barriers to vaccine uptake, particularly among underrepresented or hesitant populations. Such a multifaceted approach not only increases vaccine coverage but also fosters public trust in vaccination programs, ultimately strengthening the resilience of healthcare systems against future public health threats.^41^ By prioritizing these strategies, we aim to achieve higher vaccine acceptance and uptake, which will lead to improved health outcomes and a reduced burden of vaccine-preventable diseases.

## Data Availability

The datasets used and analyzed during the current study available from the corresponding author on reasonable request.
